# Economic Implications of Off‐Road Cycle Lanes to Increase Physical Activity and Reduce Sex and Gender Differences in the Risk of Dementia

**DOI:** 10.1002/gps.70067

**Published:** 2025-03-11

**Authors:** Magdalena Walbaum, Elisa Aguzzoli, Laura Castro‐Aldrete, Eva Cyhlarova, Antonella Santuccione Chadha, Martin Knapp

**Affiliations:** ^1^ Care Policy and Evaluation Centre London School of Economics and Political Science London UK; ^2^ Women's Brain Foundation Zürich Switzerland

**Keywords:** dementia, economic impact, off‐road cycle lane, physical activity, sex and gender differences

## Abstract

**Background:**

Dementia represents one of the greatest global health challenges. There are known risk factors that might prevent or delay nearly 50% of the different types of dementia. There are substantial differences in risk factors and progression of dementia between women and men, including engagement in regular physical activity. The study aimed to quantify the impact of increasing women's participation in physical activity with off‐road cycles and its effect on dementia incidence, relative to men and the associated health and social care costs.

**Methods:**

Our study employed a population model with secondary data analysis approach to investigate the potential economic effect of implementing off‐cycle lanes in the UK. Data were drawn from published evidence on lifetime risk of dementia relative to physical activity in men and women for the UK population, levels of physical activity in the UK, evidence on the effectiveness of off‐road cycle lanes in increasing the level of physical activity in men and women, lifetime costs of dementia in the UK. Two scenarios were compared, assuming an increase from the baseline levels of cycling of 40.3% and 114% for women and by 36.4% and 77% for men, respectively. Sensitivity analysis was conducted to account for variations in key variables.

**Results:**

Current dementia related lifetime costs were estimated at £1090.1 billion, and total lifetime costs £3326.1 billion. Under Scenario 1, dementia related lifetime costs decreased by £4.7 billion, and total lifetime costs by £0.11 billion. In Scenario 2, dementia related lifetime costs decreased by £7.0 billion, and total lifetime costs by £1.9 billion.

**Conclusion:**

Off‐road cycle lanes, especially for women, this type of structural and lifestyle intervention has the potential to bring health and economic benefits. Increased physical activity not only provides numerous health benefits, but also contributes to preventing the onset and lifetime costs of dementia.

**Summary**

- This study quantified the effects on dementia associated health and social care lifetime costs of increasing women and men’s participation in physical activity through off‐road cycle lanes.
- The implementation of off‐road cycle lanes can lead to significant reductions in dementia related lifetime costs and total lifetime costs.
- Increasing physical activity has the potential to prevent the onset of dementia and reduce its long‐term costs, delivering both health and economic benefits.
- Off‐road cycle lanes is a structural population‐level intervention that have shown to be effective in increasing cycling patterns, and reducing the sex and gender relative gap in physical activity levels.

## 1 Background

Dementia represents one of the greatest global health challenges [1, 2]. Dementia gradually impairs individuals' personal and functional abilities, increases dependency [3], and has a major effect on quality of life by progressively reducing cognitive functions [4]. The Alzheimer's Society (2023) estimated that, in 2019, there were around 900,000 people with dementia in the UK, and this number is expected to rise to 1.6 million by 2040 [5]. There is mixed evidence related to the incidence of dementia in the UK, with studies indicating a decline [6], while others have projected an increase, which would increase the burden to health and social care, and society [7].

In 2020 and 2024, the Lancet Commission identified several key modifiable risk factors associated with dementia: low education, hearing loss, traumatic brain injury, hypertension, type 2 diabetes, excess alcohol consumption, obesity, smoking, depression, social isolation, physical inactivity, air pollution and recently added LDL cholesterol [8, 9]. The Commission stated that the risk factors identified might prevent or delay nearly 50% of the different types of dementia. As global life expectancy and longevity rise, it becomes increasingly important to detect early signs of cognitive decline, address modifiable risk factors and promote life‐long healthy habits [10] to effectively reduce the incidence of dementia. Therefore, primary and secondary prevention remain essential in efforts to prevent and/or delay damage to brain function, reducing the prevalence and impacts of dementia [2, 11, 12]. There are substantial differences in risk factors and progression of dementia between women and men [13]. Women face an increased lifetime risk of developing dementia [14, 15], with nearly double the risk of developing AD compared to men, partially because of their longer life expectancy and extended exposure to dementia risk factors [16]. Sex and gender differences in dementia risks are multifaceted [15], and can act as key biological and social determinants of brain health. Thus, existing inequities in risk factors between men and women can significantly impact the incidence and progression of the condition [17].

Physical activity is associated with a reduced risk of cognitive impairment in later‐life and onset of dementia [9, 18, 19, 20] through direct mechanisms, such as improved blood flow, increased nitric oxide production leading to enhanced neurogenesis and reduced neuroinflammation, as well as indirect benefits by preventing or delaying other dementia risk factors, including high LDL cholesterol, diabetes, hypertension and obesity [9]. For example, leisure‐time physical activity has been shown to be particularly protective against Alzheimer's disease (AD) [21]. Moreover, a systematic review and meta‐analysis showed that higher levels of physical activity were associated with a 14% reduction in risk of dementia compared to lower levels of physical activity [22]. Similarly, physical exercise has been shown to improve cognitive function, with moderate‐intensity weekly exercise lasting at least 45–60 min per session associated with cognitive benefits [23]. The Whitehall Study found that more than 2.5 h of self‐reported, moderate‐to‐vigorous physical activity per week lowered dementia risk over 10 years [8]. In a 44‐year study of the Swedish population, it was found that 32% of the participants with low baseline fitness, 25% with medium fitness and only 5% with high fitness developed dementia [8]. While preventing dementia may reduce related health and social care costs, increased life expectancy exposes individuals to other conditions, which will likely require support from health and/or social care services. Further research is needed to assess long‐term savings from dementia risk‐reduction, while accounting for increasing costs from co‐occurring conditions.

Several interventions have been shown to be effective and cost‐effective in increasing physical activity. Most of the evidence has focused on individual‐level interventions and specific population groups, with more limited research on population‐level interventions [24, 25]. A review by Laine et al. found that modifying the built environment, such as introducing cycle lanes or public parks, may be the most cost‐effective strategy for increasing physical activity at the population level [24]. However, there are notable sex and gender differences in physical activity participation. A systematic review showed that, in general, men engage in higher levels of physical activity compared to women, and women are less likely to achieve regular physical activity [26]. Furthermore, sex and gender differences become evident in the rates of regular physical activity and exercise frequency among older people, as participation in leisure and outdoor activities decline with age, particularly among women [27]. Also, due to wider barriers such as perceived safety of outdoor spaces, gender roles and responsibilities within the household such as parenthood and caregiving, women’s physical activity participation can remain low across their lifespan [28, 29, 30]. In relation to cycling, several factors influence engagement, with women showing a stronger preference for routes that provide maximum separation from other motorised traffic [31, 32]. To further understand the protective role of physical activity, the present study aims to quantify the impact of increasing women's participation in physical activity with off‐road cycles by reducing dementia incidence, relative to men and the associated health and social care lifetime costs.

## 2 Methods

### 2.1 Study Design

To demonstrate the potential for an intervention that is differentially effective for women, we focused on the impact of off‐road cycle lanes in increasing women's levels of physical activity relative to men, thereby potentially reducing sex and gender differences in dementia risk and the associated health, quality of life and economic consequences. Our study employed a population model with secondary data analysis approach to investigate the potential economic effect of implementing off‐cycle lanes in the UK. Data were drawn from published evidence on lifetime risk of dementia relative to physical activity in men and women for the UK population, evidence on the effectiveness of off‐road cycle lanes in increasing the level of physical activity in men and women, and the lifetime costs of dementia in the UK. This allowed us to estimate the reduction in the prevalence of dementia for men and women with increased physical activity levels, and the overall economic impacts from the health and social care perspective. The model was populated with the UK population between 16 and 64 years old, assuming that uptake of cycling would primarily occur in this age group rather than among those aged 65 and older. It was used to explore the lifetime risk of dementia in men and women based on changes in their physical activity patterns with the introduction of off‐road cycle lanes. This allowed us to estimate gender‐specific changes in overall lifetime health and care costs, as well as dementia‐specific lifetime costs.

### 2.2 Model Inputs

Parameters used in the model are shown in Table 1. Data on risk of dementia for men and women associated with physical inactivity for the English population were extracted from Van Baal et al. [33]. That study developed a dynamic Markov simulation model to estimate the associations between levels of physical activity, incidence of dementia, mortality and the lifetime use of health care and social care in England. The model included three states: no dementia, dementia, and death. The no dementia and dementia states were stratified by gender, age and physical activity level. The model simulated annual changes in the population incidence of dementia and mortality over time, associated with variations in levels of physical activity, estimating the years living with dementia and the associated costs. Further details on the methods of the Markov model can be found in the published article [33].

**TABLE 1:** Parametres of the model.

Variable	Estimate	Source
Relative risk of dementia^a^		
Low activity	0.65	Van Baal et al. [33]
Some activity	0.65	
Meet aerobic guidelines	0.62	
Proportion of people cycling 2–5 km		
Women	0.026	Grudgings et al. [32]
Men	0.087	
Increase in cycling with off‐road cycle lanes		
Women	40.3%–114.5%	Parker et al. [34]
Men	36.4%–77.1%	Vasilev et al. [35]

^a^ Reference category inactive.

Our population level model used estimate for the proportion of adults cycling at least once a week in England was extracted from the Census 2021 data and the absolute and relative gender gap in cycling patterns between men and women in England were taken from Grudgings et al. [32]. Data on the effectiveness of off‐cycle lanes in increasing cycling was taken from Parker et al. [34] and Vasilev et al. [35], showing an increase by 114.5% for women and 77.1% for men, and 40.3% for women and 36.4% for men, respectively. Estimates of the English population were extracted from the Census 2021 data [36].

Levels of physical activity used in our model were based on the current level of physical activity in the English population and were extracted from the Health Survey for England 2021 and are shown in Table 2. We focused our analyses on the population between 16 and 64 years of age given that this group of the population would likely benefit the most with the availability of off‐road cycle lanes and would have a direct impact on the risk of developing dementia. All data related to costs are presented in Table 3. Data on costs of dementia were extracted from Van Baal et al. [33]. Data on costs related to unpaid carers were extracted from Livingston, Barber et al. [37]. Data on health care and social care costs for other conditions were extracted from Van Baal et al. [33] (Table 3). All costs were adjusted to 2024 prices.

**TABLE 2:** Levels of physical activity of the English population.

	Age group (years)						
Activity level	16–24	25–34	35–44	45–54	55–64	65–74	75+
Women							
Meets aerobic guidelines	57	66	66	67	58	55	35
Some activity	14	14	14	13	11	12	13
Low activity	4	6	5	4	4	7	5
Inactive	24	15	15	15	27	26	47
Men							
Meets aerobic guidelines	72	78	78	72	69	65	41
Some activity	8	7	8	10	10	11	11
Low activity	1	4	3	2	2	4	4
Inactive	18	11	11	16	20	19	43

*Note:* All values are percentages (%). Columns may not sum to exactly 100% due to rounding approximations Activity levels are defined as: Meets aerobic guidelines: At least 150 min moderately intensive physical activity (MPA) or 75 min vigorous activity (VPA) per week (pw) or an equivalent combination of these, in episodes of 10 min or more. Some activity: 60–149 min MPA pw or 30–74 min VPA pw or an equivalent combination of these, in episodes of 10 min or more. Low activity: 30–59 min MPA pw or 15–29 min VPA pw or an equivalent combination of these, in episodes of 10 min or more. Inactive: Less than 30 min MPA pw or less than 15 min VPA pw or an equivalent combination of these, in episodes of 10 min or more.

**TABLE 3:** Current lifetime costs of dementia per person (£, 1000s).

	Inactive	Low activity	Some activity	Meets aerobic guidelines
Women				
Health care costs, dementia	19.7	13.8	14.3	14.1
Health care costs, other diseases	41.2	44.2	44.8	45.7
Social care costs, dementia	9.2	6.5	6.6	6.6
Social care costs, other diseases	7.4	8.8	9.1	9.5
Costs of unpaid carers	2.23	1.63	1.63	1.63
Men				
Health care costs, dementia	19.2	13.3	13.7	13.5
Health care costs, other diseases	34.1	37.3	38	39
Social care costs, dementia	8.9	6.2	6.4	6.3
Social care costs, other diseases	3.5	4	4.2	4.3
Costs of unpaid carers	2.18	1.58	1.58	1.58
Total costs women	79.76	74.93	76.43	77.53
Total costs men	67.88	62.38	63.88	64.68

*Note:* Costs were extracted from Van Baal et al. [33] and Livingston, Barber et al. [37].

Current lifetime population costs of dementia for people aged between 16 and 64 years are presented in Table 3. The estimates of costs of unpaid care were calculated using the years with dementia for the different physical activity levels [33] and the costs of unpaid care of people with dementia per year, per person [37] (Table 3). Based on the Census 2021 data, 12% of the adult population in England cycles at least once a week. The proportion of women and men cycling 2–5 km is presented in Table 4.

**TABLE 4:** Cycling patterns by men and women.

	Baseline	Scenario 1	Scenario 2
Women	2.79	4.0	6.0
Men	8.89	12.1	15.8
Absolute gender gap	6.1	8.1	9.8
Relative gender gap	3.3	3.0	2.6

*Note:* All values are percentages (%), except for the relative gender gap, which it's expressed as a ratio rather than a percentage change. Percentages of baseline were calculated based on the Census data 2021 of cycling at least once a week and the relative and absolute gap between women and men extracted from Grudgings et al. [32] Scenario 1 assumed an increase by 40.3% for women and by 36.4% for men from the baseline levels presented in Table 4. Scenario 2 assumed an increase by 114% for women and by 77% for men from the baseline levels.

Based on the definitions of physical activity used in the Health Survey for England (2021), we assumed that a proportion of inactive individuals, those with low activity levels and those with some activity would increase their levels of physical activity with the implementation of off‐road cycle lanes by one level, for example, inactive people would increase to low activity levels. The proportion of people that meet the recommendations of physical activity would remain in that level. We assumed an increase in the current proportion of cycling in England [32] based on the estimates of effectiveness of off‐cycle lanes in increasing cycling reported by Parker et al. [34] and Vasilev et al. [35]. We present two scenarios of different rates of change in physical activity levels in men and women based on the proportion of cycling of women and men. The first scenario assumed an increase by 40.3% for women and by 36.4% for men, and the second scenario assumed an increase by 114% for women and by 77% for men from the baseline levels, respectively. The estimated cycling proportions are shown in Table 4.

We calculated the total number of people by level of physical activity in the baseline scenario based on the current levels of physical activity (Table 2). We then assumed an increase in the proportion of people engaging in physical activity with the implementation of off‐road cycle lanes (Table 4) to estimate the changes in the number of people by level of physical activity in Scenario 1 and Scenario 2. Finally, we multiplied the total number of people in each scenario by the lifetime costs of dementia (Table 3).

### 2.3 Sensitivity Analysis

We conducted probabilistic sensitivity analysis (PSA) with 1000 simulations to account for variations in base case model inputs. The PSA was undertaken whereby different probability distributions were associated with each parameter of the model depending on the data, using the mean value and the 95% confidence interval of the estimate. For the costs data, we assumed a baseline cost ± 20% variation in the mean value to calculate the 95% confidence interval used in the PSA. The choice of the types of distributions was according to standard practice and to the nature of each parameter [38, 39]. For probabilities, Beta distributions were used; and for costs, Gamma distribution. We also analysed two different scenarios of the impact of off‐road cycle lanes by adjusting the estimated rate of change in physical activity levels in men and women based on the different proportions of increase in cycling uptake reported by Parker et al. [34] and Vasilev et al. [35]. Microsoft Excel Office 365 were used to construct the model and the different scenarios.

## 3 Results

The results show a change in the dementia related lifetime costs and total lifetime costs for individuals aged between 16 and 64 years, resulting from increased physical activity levels due to the implementation of off‐road cycle lanes and based on the current English population in these age groups (Table 5).

**TABLE 5:** Population lifetime costs by different physical activity levels with off‐road cycle lanes (£, billions).

	Physical activity level	Baseline	Scenario 1	Scenario 2
Dementia related lifetime costs				
Women	Inactive	140.87	135.34	132.55
	Low activity	24.37	27.38	28.83
	Somewhat active	71.50	69.80	68.78
	Meets aerobic guidelines	332.03	334.60	336.09
	Total	568.77	567.12	566.25
Men	Inactive	105.74	93.06	89.14
	Low activity	11.61	19.13	21.38
	Somewhat active	43.70	39.95	38.73
	Meets aerobic guidelines	361.07	366.99	368.44
	Total	522.13	519.13	517.69
Total lifetime costs				
Women	Inactive	360.32	346.17	339.02
	Low activity	83.11	93.36	98.31
	Somewhat active	242.20	236.46	233.02
	Meets aerobic guidelines	1150.85	1159.76	1164.90
	Total	1836.48	1835.75	1835.25
Men	Inactive	236.69	208.30	199.53
	Low activity	34.29	56.50	63.13
	Somewhat active	128.58	117.55	113.96
	Meets aerobic guidelines	1090.01	1107.85	1112.25
	Total	1489.57	1490.20	1488.88

*Note:* Total numbers may vary slightly due to rounding approximations. Activity levels are defined as: Meets aerobic guidelines: At least 150 min moderately intensive physical activity (MPA) or 75 min vigorous activity (VPA) per week (pw) or an equivalent combination of these, in episodes of 10 min or more. Some activity: 60–149 min MPA pw or 30–74 min VPA pw or an equivalent combination of these, in episodes of 10 min or more. Low activity: 30–59 min MPA pw or 15–29 min VPA pw or an equivalent combination of these, in episodes of 10 min or more. Inactive: Less than 30 min MPA pw or less than 15 min VPA pw or an equivalent combination of these, in episodes of 10 min or more. Scenario 1 assumed an increase by 40.3% for women and by 36.4% for men from the baseline levels presented in Table 4. Scenario 2 assumed an increase by 114% for women and by 77% for men from the baseline levels.

Total costs by gender from Table 5 are included in Table 6, which shows the savings in dementia related lifetime costs and total lifetime costs associated with changes in physical activity levels for the UK population aged16 to 64 years, resulting from the implementation of off‐road cycle lanes. Under the baseline scenario, dementia related lifetime costs were estimated at £1090.1 billion, and total lifetime costs were £3326.1 billion. Under Scenario 1, population dementia‐related lifetime costs decreased by £4.7 billion to £1086.3 billion, and total lifetime costs decreased by £0.11 billion to £3325.9 billion. In Scenario 2, assuming a larger increase in physical activity (114% for women and 77% for men), dementia related lifetime costs decreased by £7.0 billion to £1083.9 billion, and total lifetime costs reduced by £1.9 billion to £3324.1 billion.

**TABLE 6:** Difference in lifetime costs related to changes in physical activity levels with off‐road cycle lanes (£, billions).

	Baseline	Scenario 1	Scenario 2
Dementia related lifetime costs			
Women	568.77	567.12	566.25
Men	522.13	519.13	517.69
Total	1090.91	1086.25	1083.94
Difference		−4.65	−6.96
Total lifetime costs			
Women	1836.48	1835.75	1835.25
Men	1489.57	1490.20	1488.88
Total	3326.05	3325.95	3324.13
Difference		−0.11	−1.93

*Note:* Total numbers may vary slightly due to rounding approximations. Scenario 1 assumed an increase by 40.3% for women and by 36.4% for men from the baseline levels presented in Table 4. Scenario 2 assumed an increase by 114% for women and by 77% for men from the baseline levels.

## 4 Discussion

Several observational studies have shown significant associations between risk factors and dementia; however, causal relationships between these factors are still under examination [4]. While much of the available evidence is observational, prevention interventions have the potential to generate positive outcomes across various health aspects, and address individuals' physical, psychological and overall wellbeing.

The economic analysis quantified the potential benefits of off‐road cycle lanes in reducing the prevalence of dementia related to physical activity, and the dementia related lifetime costs and overall lifetime costs resulting from changes in the physical activity levels of the English population aged between 16 and 64 years. While dementia prevention may lower related health and social care costs, increased life expectancy exposes individuals to other conditions, potentially increasing long‐term care costs. As a result, the reduction in overall lifetime costs is smaller than the reduction in dementia‐related costs. However, despite this difference, there are still overall cost savings. Structural arrangements such as off‐road cycle lanes can be effective in proportionately increasing the number of women who choose cycling as a mode of transportation, as shown by the evidence [31, 35, 40]. In this study, we did not include the costs associated with implementing off‐road cycle lanes or other infrastructure changes, which could influence the overall impact of these initiatives on dementia‐related health and care costs. While the focus was on the potential reduction in lifetime health and care costs related to dementia resulting from increased physical activity, it is important to recognise that the initial investment in cycle lanes, along with ongoing maintenance and safety measures, could influence the results. Further research should investigate these infrastructure‐related costs to assess the cost‐effectiveness of promoting physical activity through cycling infrastructure in the context of dementia prevention. Nevertheless, evidence suggests that investments in cycling facilities are likely to increase active commuting practices by cycling to work [41], and they have been estimated to be cost‐effective or even cost‐saving in the long‐term even without including the benefits of reducing dementia risk [2].

Affordable actions addressing safety, equality and accessibility need to be considered as a public health priority to reduce sex and gender differences in the prevention risk factors for dementia. The FINGER trial represents a recent example of a multidomain intervention which showed signs of improvement in cognitive functioning in the older population [11]. Although sex and gender differences were not assessed, this trial showed that a prevention strategy targeting multiple dementia risk factors simultaneously could result in better outcomes [11] and to be cost‐effective [42]. Furthermore, to implement behavioural interventions such as healthy lifestyles, it is strongly agreed that sex and gender differences must be considered for successful outcomes [16].

When implementing population‐level interventions, there should be a strong emphasis on equity, accessibility, affordability and low agency to achieve large‐scale impact. Moreover, there is a need to focus on adapting and changing the environment to consequently change the choices that people make by default [43]. Furthermore, because dementia risk factors are modifiable throughout the lifespan, it becomes fundamental to adopt a life‐course approach to build long‐term cognitive reserve, and to prevent and delay dementia onset through everyday activities.

Sex and gender differences in physical activity, especially during adulthood, are partly influenced by roles and responsibilities within family households, as well as safety concerns. Addressing safety concerns can be a viable solution to narrow the sex and gender gap in physical activity participation. Increasing women's confidence and ensuring access to safe environments for exercise would enable women to have more equal opportunities and easier access to an active lifestyle. Educational interventions on the health benefits of physical activity and its role in delaying dementia onset and cognitive decline can prove ineffective if the context and environment in which women live are hostile and unsafe. Therefore, designing and building environments inclusive of all protective characteristics, such as safety and accessibility, particularly for older people and women, have the potential to be viable solutions.

Some studies have examined the effects of improving cycling structures as a population‐level intervention to increase physical activity within the population. A study conducted in Australia showed that the most important enablers to cycling included having an off‐road bike path (66%), as well as individuals being committed to improving their personal physical health (65%) and reducing their environmental impact (57%) [44]. A significantly higher proportion of women reported specific safety concerns about cycling alongside motor vehicle traffic compared to men [45].

These sex and gender differences in cycling patterns and are well‐documented in several studies [46, 47, 48]. In the US, men's bicycle trips exceeded women's by a ratio of 2 to 1—a consequence of individual, social and physical factors [47]. Similarly, men were more likely to cycle for recreation and for transport compared to women, and for longer time [46]. Importantly, men were more likely to cycle on‐road, while women were more likely to cycle off‐road [31, 46] due to increased safety and accessibility. A study based in six European countries demonstrated that women experienced higher discomfort and distress than men when cycling in mixed traffic [48]. Providing off‐road cycle lanes in UK cities can significantly increase cycling patterns and times [49], contributing to an increase in overall physical activity [41]. The evidence shows a higher benefit for women relative to men, and thus reducing the sex and gender gap in physical activity participation and the health and economic impact of dementia. Given that fewer women feel safe in cycling and engaging in physical activities under current conditions, improving road safety can be a highly effective intervention to increase cycling rates [50].

## 5 Strengths and Limitations

This study has several strengths. We employed a population‐level modelling approach using well‐established secondary data sources to estimate the potential economic impacts of gender‐sensitive interventions such as off‐road cycle lanes in reducing dementia risk, emphasising the potential economic benefits of increasing cycling uptake through infrastructure improvements.

The inclusion of gender‐specific data allows exploration of differential effects of cycling uptake between men and women, offering valuable insights into gender disparities in physical activity and dementia‐related outcomes. Moreover, the study integrates data from multiple robust sources, including national datasets such as the Population Census 2021 and the Health Survey for England 2021, as well as peer‐reviewed literature on the association between physical activity and dementia risk. Evidence on dementia‐related outcomes and costs was derived from a well‐conducted dynamic Markov simulation model, which estimates lifetime health and social care costs for dementia in England.

Furthermore, scenario analyses were undertaken to assess the impact of different assumptions regarding the effectiveness of off‐road cycle lanes in increasing cycling uptake among men and women, thereby providing a comprehensive evaluation of the potential effects of this intervention. Additionally, we incorporated probabilistic sensitivity analysis, strengthening the robustness of the results by accounting for uncertainty in key model parameters.

Nevertheless, the study is subject to several limitations. While the model is informed by high‐quality published data, future projections would require updating as new evidence emerges to calibrate and validate estimates. The model assumes that the relationship between cycling uptake and physical activity levels follows a uniform stepwise increase, which may not fully capture the complexities of real‐world behavioural changes. Furthermore, the study does not account for potential barriers to cycling uptake, such as infrastructure accessibility, safety concerns, or socio‐economic disparities, which may influence engagement differently across population subgroups.

Additionally, this study does not incorporate the costs associated with implementing off‐road cycle lanes, which is a critical factor when considering large‐scale deployment across diverse communities. The model is restricted to individuals aged 16–64 years, thereby excluding potential benefits for older adults, who may also experience health improvements from increased physical activity. Although the study considers gender differences in dementia risk, other intersectional factors, including ethnicity, socio‐economic status and comorbidities, were not explicitly modelled.

## 6 Conclusions

Dementia prevention awareness should be widely advocated, both at the national level and within local communities. Services targeting dementia should be easily accessible and so that they can be reached by everyone. Effective action will require policy change at all levels of governance [4]. Because awareness of dementia risk‐reduction is low, knowledge about modifiable risk factors and prevention mechanisms is key to improving and achieving healthy ageing [8]. To effectively promote physical activity, population‐level risk reduction approaches should be impactful, far‐reaching and cost‐effective. These approaches should target the most vulnerable individuals and consider sex and gender differences [3, 4].

Structural changes in urban planning and local settings are needed to create more ‘sex and gender‐friendly’ communities. This includes not only creating spaces for socialisation at the community level, but also implementing policies to ensure a safer environment for women to exercise and be physically active. As demonstrated by the physical activity case study and the positive outcomes shown with off‐road cycle lanes, especially for women, this type of structural and lifestyle intervention has the potential to bring health and economic benefits [10]. Increased physical activity not only provides numerous health benefits, but also contributes to preventing the onset and lifetime costs of dementia.

## Conflicts of Interest

A.S.C. is founder of the Women's Brain Foundation and Vice President at Euresearch. All other authors declare no conflict of interests.

## Data Availability

The authors have nothing to report.
